# Supplementary material for: Second waves, social distancing, and the spread of COVID-19 across the USA
Source: Wellcome Open Res. 2021 Mar 15;5:103. Originally published 2020 May 26. [Version 3] doi: 10.12688/wellcomeopenres.15986.3 (PMC8063524; doi:10.12688/wellcomeopenres.15986.3)
Supplement: Peer review history

# Peer review: Reviewer response for version 3

**DOI:** 10.21956/wellcomeopenres.18424.r43044

**Published:** 2021-04-19

**Authors:** Andrei R. Akhmetzhanov

**Affiliations:** 1 Global Health Program, National Taiwan University College of Public Health, Taipei City, Taiwan

I think the manuscript looks good. I would still consider it as a proof of concept, because inclusion of only 8 most-hit states of the US can be regarded as a limiting factor. I think that the travelers were allowed to travel between all states, therefore the system of 8 states does not represent a closed system. I recommend approval of the manuscript and have only an advice to correct Table 1 if possible.

 Table 1: There could be some confusion about the units of the variance. For example, the variance for "contagious period (days)" is likely shown as 1/64 days^(-2), but then the description is wrong, because tau is in days, variance is in days^(-2). Am I correct? I am more wondering, because the variance of 1/64 looks a bit too narrow for me if I compare it with the mean of 4 days. (The same remark holds for other rows of the table)

Is the rationale for developing the new method (or application) clearly explained?

No

Is the description of the method technically sound?

Partly

Are the conclusions about the method and its performance adequately supported by the findings presented in the article?

Partly

If any results are presented, are all the source data underlying the results available to ensure full reproducibility?

Partly

Are sufficient details provided to allow replication of the method development and its use by others?

Partly

Reviewer Expertise:

Infectious diseases modeling

I confirm that I have read this submission and believe that I have an appropriate level of expertise to confirm that it is of an acceptable scientific standard.

# Peer review: Reviewer response for version 3

**DOI:** 10.21956/wellcomeopenres.18424.r43043

**Published:** 2021-03-24

**Authors:** Alexander Zarebski

**Affiliations:** 1 Department of Zoology, University of Oxford, Oxford, UK

Between edits to the manuscript and their written response, the authors have addressed all of my concerns and I am happy to approve this manuscript.

Is the rationale for developing the new method (or application) clearly explained?

Yes

Is the description of the method technically sound?

Partly

Are the conclusions about the method and its performance adequately supported by the findings presented in the article?

Partly

If any results are presented, are all the source data underlying the results available to ensure full reproducibility?

Yes

Are sufficient details provided to allow replication of the method development and its use by others?

Partly

Reviewer Expertise:

Mathematical epidemiology and computational statistics.

I confirm that I have read this submission and believe that I have an appropriate level of expertise to confirm that it is of an acceptable scientific standard.

# Peer review: Reviewer response for version 2

**DOI:** 10.21956/wellcomeopenres.18314.r42518

**Published:** 2021-02-24

**Authors:** Alexander Zarebski

**Affiliations:** 1 Department of Zoology, University of Oxford, Oxford, UK

Thank you for addressing the points raised in my previous review. For the most part your responses have satisfied me, although I still have a few reservations. Moreover, in re-reading your work I spotted a couple of other things that I would like you to address, I apologise for not catching them in the first review, I hope they are still of some use. The significant point remaining from the first review is:

 1. You have clearly acknowledged the limitation of assuming New York as the seeding location, thank you. You have not assessed the impact of this assumption though. It is hard to guess how significant it is, yet given the structure in the selected connectivity matrix it seems worth pursuing. That said, I also pointed out that the manuscript is already large so addressing this would need to be done with care.

 Things not mentioned in the previous review that should have been, include the following:

 2. You have only used data from the eight states with the greatest number of cases, suggesting that they are the most informative. However, I suspect that ignoring the states which have not seen substantial growth means you have introduced a type of observation bias into you data. You have only generated estimates using the most extreme data points, those where local epidemics took hold. This could lead to bias in the estimates.

 3. Footnote 1 suggests that this work is a "proof of concept" and primarily "didactic", and in some parts of the Conclusion you seem to suggest that the output of this work is not a concrete prediction so much as an exploration of model output. That is fine and important. Given the current situation we must be very careful though, and given the amount of material in the text, it can be difficult to work out what is intended as statements about plausible outcomes of the pandemic in the USA and what the outcomes of the modelling are. Please keep this in mind during any further revisions. As previously mentioned, the manuscript is long, and I suspect trimming it down may partially solve this issue.

Is the rationale for developing the new method (or application) clearly explained?

Yes

Is the description of the method technically sound?

Partly

Are the conclusions about the method and its performance adequately supported by the findings presented in the article?

Partly

If any results are presented, are all the source data underlying the results available to ensure full reproducibility?

Yes

Are sufficient details provided to allow replication of the method development and its use by others?

Partly

Reviewer Expertise:

Mathematical epidemiology and computational statistics.

I confirm that I have read this submission and believe that I have an appropriate level of expertise to confirm that it is of an acceptable scientific standard, however I have significant reservations, as outlined above.

# Response

**Published:** 2021-03-04

**Authors:** Adeel Razi

**Affiliations:** Monash University, Australia

Dear Dr. Zarebski,

 We would like to thank you for the time and effort you have spent reviewing our manuscript for the second time. Below are the replies to your additional comments. We hope these revisions are what you had in mind.

*1. You have clearly acknowledged the limitation of assuming New York as the seeding location, thank you. You have not assessed the impact of this assumption though. It is hard to guess how significant it is yet given the structure in the selected connectivity matrix it seems worth pursuing. That said, I also pointed out that the manuscript is already large so addressing this would need to be done with care.*

 We agree entirely. In a normal situation, we would certainly include a brief analysis of changing the seed location. However, this paper is now a legacy contribution. In other words, to go back to the model structure used last year (which was only fit for purpose in modelling the first wave) would be a purely academic exercise. Practically speaking, we are still actively modelling the subsequent waves in the UK with third or fourth generation models, which is occupying all of our available time at the moment. We have therefore tried to deal with this point by being more explicit in accommodating your point in our previous response:

 “We note that the model reduction identified a model where migration was restricted to New York and remaining states. A natural question is whether this reflects the assumption that the epidemic was seeded in New York. This kind of question suggests a new model space in which models that have multiple seeds are compared. More generally, any initial condition or prior assumption of this sort is part of the generative model and can be evaluated using Bayesian model comparison. The application of Bayesian model reduction in this setting would require a full model in which the epidemic was seeded in multiple regions. Reduced models with then comprise seeds in subsets of States.

**Although not illustrated here, the ensuing differences in model evidence would then score the ‘significance’ of assuming a single seed region. In other words, it is possible that a single region model affords a more parsimonious (higher evidence) explanation for the data. Conversely, if a multiple seed region model had a substantially greater evidence, the simplifying assumption of a New York seed would need to be seriously questioned. In terms of a parametric or quantitative characterization of structural differences between models, one generally uses a sensitivity or contribution analysis; in which variations in certain outcomes are expressed as a partial derivative of the parameters that define one model in relation to another** **(Friston et al., 2017)** **.** **”**

*2. You have only used data from the eight states with the greatest number of cases, suggesting that they are the most informative. However, I suspect that ignoring the states which have not seen substantial growth means you have introduced a type of observation bias into your data. You have only generated estimates using the most extreme data points, those where local epidemics took hold. This could lead to bias in the estimates.*

 That is an important point. We have added the following in the discussion to pursue the implications of this observation:

 “We restricted our model inversion to data from States with the greatest number of cases. Technically, this reflects a choice of the data features that provide the most information about the unknown parameters of the epidemiological model. There are established approaches to feature selection based upon the principles of optional Bayesian design (Lindley, 1956; MacKay, 1992). In the setting of dynamic causal modelling, these principles can be leveraged, in a simple way, to compare the informativeness of one set of data relative to another—based upon the degree to which prior beliefs are updated to posterior beliefs (Zeidman et al., 2019). However, Bayesian model comparison cannot be used to optimize data feature selection. This is because one has to compare different models of the same data, as opposed to the same model of different data.

 Could selecting one set of data (e.g., eight American States) lead to an observation bias? In principle, it cannot: in the sense that the maximum *aposteriori* estimates of model parameters can only be biased by prior beliefs (usually by being ‘shrunk‘ to the prior mean). Having said that, the inferences based upon posteriors over model parameters – and marginal likelihoods over models – are restricted to the particular States in question and cannot be generalized to all States that could have been modelled.”

*3. Footnote 1 suggests that this work is a "proof of concept" and primarily "didactic", and in some parts of the Conclusion you seem to suggest that the output of this work is not a concrete prediction so much as an exploration of model output. That is fine and important. Given the current situation we must be very careful though, and given the amount of material in the text, it can be difficult to work out what is intended as statements about plausible outcomes of the pandemic in the USA and what the outcomes of the modelling are. Please keep this in mind during any further revisions. As previously mentioned, the manuscript is long, and I suspect trimming it down may partially solve this issue.*

 Yes, this is an important point and speaks to the legacy aspect of this contribution, which was primarily procedural. Hopefully, the above additions will make it clear that this contribution is about Bayesian procedures to make the most of epidemiological data – not to provide any definitive predictions or conclusions (especially, given this model was only concerned with how a first wave of infection spreads among communities or states. Furthermore, your point above – and this response – will be in the public domain.

**Reference:**

 Friston, K. J., Preller, K. H., Mathys, C., Cagnan, H., Heinzle, J., Razi, A., & Zeidman, P. (2017). Dynamic causal modelling revisited. *Neuroimage*. doi:10.1016/j.neuroimage.2017.02.045

 Lindley, D. V. (1956). On a Measure of the Information Provided by an Experiment. *Ann. Math. Statist., 27*(4), 986-1005. doi:10.1214/aoms/1177728069

 MacKay, D. J. C. (1992). Information-Based Objective Functions for Active Data Selection. *Neural Computation, 4*(4), 590-604. doi:10.1162/neco.1992.4.4.590

 Zeidman, P., Kazan, S. M., Todd, N., Weiskopf, N., Friston, K. J., & Callaghan, M. F. (2019). Optimizing Data for Modeling Neuronal Responses. *Front Neurosci, 12*(986). doi:10.3389/fnins.2018.00986

# Peer review: Reviewer response for version 1

**DOI:** 10.21956/wellcomeopenres.17532.r40780

**Published:** 2020-11-02

**Authors:** Andrei R. Akhmetzhanov

**Affiliations:** 1 Global Health Program, National Taiwan University College of Public Health, Taipei City, Taiwan

The present model in the paper is quite complex and include many different factors. What confuses me about the work is fitting the between-States mobility pattern to the data. It is a bit strange for me. First, because the mobility is what can be directly measured. Second, considering the rates of mobility as the parameters for the fit may give misleading results as it would be heavily affected by the prior choice of the underlying epidemiological model. I am also afraid that the used framework of the variational Bayes is unable to give strict results that can be validated and believed to be the true ones--close to the reality. At least, I was not able to find the strong evidence of this in the paper. Because of that, the present results can be considered only with some degree of speculation.

 About the abstract:

 The first two sentences of the abstract can be confusing. The first sentence says about one causal mode, while the second sentence says about combining several of *these* models. I am also confused about whether the authors consider only US or the whole North and South America as stated in the title.

Is the rationale for developing the new method (or application) clearly explained?

No

Is the description of the method technically sound?

Partly

Are the conclusions about the method and its performance adequately supported by the findings presented in the article?

Partly

If any results are presented, are all the source data underlying the results available to ensure full reproducibility?

Partly

Are sufficient details provided to allow replication of the method development and its use by others?

Partly

Reviewer Expertise:

Infectious diseases modeling, outbreak analysis

I confirm that I have read this submission and believe that I have an appropriate level of expertise to confirm that it is of an acceptable scientific standard, however I have significant reservations, as outlined above.

# Response

**Published:** 2021-01-31

**Authors:** Adeel Razi

**Affiliations:** Monash University, Australia

Dear Prof. Akhmetzhanov,

 We would like to thank you for the time and effort you have spent reviewing our manuscript. Below are the replies to the comments. We hope these revisions are what you had in mind.

- *The present model in the paper is quite complex and include many different factors. What confuses me about the work is fitting the between-States mobility pattern to the data. It is a bit strange for me. First, because the mobility is what can be directly measured. Second, considering the rates of mobility as the parameters for the fit may give misleading results as it would be heavily affected by the prior choice of the underlying epidemiological model.*

This is an interesting observation. We have taken this point in development along the following lines (in the Conclusion):

 “In this paper, we estimated the effective connectivity or population fluxes between States that, in principle, can be measured empirically. One might ask why not use proxies for exchange between regions based upon transport or mobility data? A useful answer here is that the use of proxies rests on an implicit hypothesis; namely, that the chosen proxy provides an informative constraint on the latent variable in question. Crucially, one can test this hypothesis by comparing two models with and without prior constraints based upon proxy measurements. An interesting example of this in imaging neuroscience is the use of structural connectivity measurements from tractography data as priors on effective connectivity (Sokolov et al., 2019). In short, if a model with priors (e.g., mobility data) has more evidence than a model without, one can claim that the proxies are licensed in a useful way.”

  

- *I am also afraid that the used framework of the variational Bayes is unable to give strict results that can be validated and believed to be the true ones--close to the reality. At least, I was not able to find the strong evidence of this in the paper. Because of that, the present results can be considered only with some degree of speculation.*

These are good points that pertain to any Bayesian inference, especially at the level of model comparison. There are many issues that one could discuss about variational inference in this regard. For example, we now highlight a couple of the limitations as follows (in the Conclusion):

 “We have noted some of the limitations of dynamic causal modelling—and more generally variational Bayes—above. Two key limitations are (i) the overconfidence problem and (ii) the robustness of the Laplace assumption. The overconfidence problem derives from a failure to explicitly account for conditional dependencies among the factors that constitute the mean field approximation that defines variational procedures. This is a ubiquitous problem in any variational approach that tries to simplify model inversion by breaking the problem into a series of marginal posteriors. Generally, one just acknowledges the overconfidence problem or, as in this application of dynamic causal modelling, inflate the credible intervals such that they encompass the data points. The robustness of the Laplace assumption speaks to the discrepancy between the form of the true and approximate posterior that has two implications. First, one is committed to unimodal Gaussian approximations to the posterior density that might be multimodal. The second implication is that the assumed (quadratic) form for the objective function may be violated, creating local minima. The solution to these problems generally rests on finessing nonlinearities by using different functional forms for the likelihood or priors. For example, using log transforms as in the current application. The very existence of local minima and failures of convergence are often used as a diagnostic to suggest that the generative model (and implicit formal priors) need attention. As illustrated above, different generative models can then be optimized in relation to their free energy.”

 In responding to the other reviewer, we also try to make it clear that Bayesian model reduction can be used to convert a concern about the appropriateness of a model into a question and then answer that question formally in terms of the evidence at hand. For example:

 “Here we note that the model reduction identified a model where migration was restricted to New York and remaining states. A natural question is whether this reflects the assumption that the epidemic was seeded in New York. This kind of question suggests a new model space in which models that have multiple seeds are compared. More generally, any initial condition or prior assumption of this sort is part of the generative model and can be evaluated using Bayesian model comparison. The application of Bayesian model reduction in this setting would require a full model in which the epidemic was seeded in multiple regions. Reduced models with then comprise seeds in subsets of States. We do not pursue this analysis here but note that this is an interesting question.”

  

- The first two sentences of the abstract can be confusing. The first sentence says about one causal mode, while the second sentence says about combining several of *these* models.

We have amended the abstract wording to “... several instantiations of this (epidemic) model to create a (pandemic) model …”

- *I am also confused about whether the authors consider only US or the whole North and South America as stated in the title.*

We have amended the title to “... across the USA.”

**References:**

 Sokolov, A. A., Zeidman, P., Erb, M., Ryvlin, P., Pavlova, M. A., & Friston, K. J. (2019). Linking structural and effective brain connectivity: structurally informed Parametric Empirical Bayes (si-PEB). *Brain Struct Funct, 224*(1), 205-217. doi:10.1007/s00429-018-1760-8.

# Peer review: Reviewer response for version 1

**DOI:** 10.21956/wellcomeopenres.17532.r39184

**Published:** 2020-07-14

**Authors:** Alexander Zarebski

**Affiliations:** 1 Department of Zoology, University of Oxford, Oxford, UK

Friston *et al.* extend their previous epidemic model into a metapopulation of COVID-19 transmission across eight states in the US. The resulting estimates (informed by time series of confirmed cases and deaths curated by the team at Johns Hopkins University) are then used as a starting point for considering longer-term outcomes of the epidemic such as the occurrence of a secondary wave due to a loss of immunity and the need for ongoing intervention measures if a vaccine cannot be developed. The effects of social distancing are included in the model as a sigmoidal function of the prevalence. This is an interesting approach and I would like to see it developed further. A substantial amount of thought has gone into developing the generative model.

 The term "dynamic causal model" may be unfamiliar to some readers (it was to me). I understand that it refers to a hidden Markov model: in this case, a differential equation (DE) model of the epidemic where the data is assumed to be a random variable conditional upon that epidemic. Although the exact definition does not seem essential, I point this out because some of the language used may be unfamiliar but as far as I can tell the constructions are all the familiar ones from mathematical epidemiology.

 The authors should be commended for the substantial efforts put into making this analysis reproducible with good documentation and by referencing the commit of the JHU repository for the data they used. Although on a fresh installation of Octave version 4.0.0 I was not able to reproduce the computation, even after following the Octave specific instructions regarding the `importdata` function. The data appeared to be read in successfully, but subsequently, an indexing error was hit and I was not able to debug further. Please note that I have not used Octave/MATLAB for several years so there may have been something obvious I was missing.

 There are several (potentially conflicting) points I would like to see addressed that I suspect would improve this manuscript.

1. The text is very conversational, which is pleasant to read, but which makes it difficult to get a strong grasp on the details. I think it would be much easier to understand the work if the text was heavily edited for brevity.
2. The model reduction has left a model in which there is only migration between New York and the other states. I worry that result may be driven entirely by the assumed initial condition in which the epidemic is only seeded in New York (a dubious assumption). I would like to see that the connectivity is robust to changes in the initial condition as I suspect allowing for importation into all the considered states will affect this.
3. I could not see whether it was the weekly number of cases/deaths or the weekly cumulative number of cases/deaths that were being used. The use of cumulative counts is questionable if one is modelling the observations as conditionally independent. Having a more explicit definition of the likelihood function would probably resolve my misunderstanding. For details of the model (before the inclusion of spatial structure) the reader is directed to a previous manuscript which seemed to suggest it was not the cumulative counts used, but then the cumulative counts are frequently referenced in the text as the data that the model is being compared to.
4. There are comments regarding the limitations of the work scattered throughout the text. I think it would be useful to have a couple of paragraphs towards after the results section spelling these out in one place. In particular, the results "are just model predictions and should not be taken literally. They should be read as the kind of predictions that can be made with suitable modelling."
5. In my (very limited) experience with variational Bayes methods, I have found the technique to be fiddly. I would like to see more evidence to suggest that the Laplace approximation is reasonable in this setting (i.e., that there is sufficient information to have a posterior that is approximately Gaussian). Ideally, one would re-run some of the inference using something such as MCMC to check that the resulting posterior samples conform to something vaguely Gaussian. Are there some sort of diagnostics that could be applied here?

Is the rationale for developing the new method (or application) clearly explained?

Yes

Is the description of the method technically sound?

Partly

Are the conclusions about the method and its performance adequately supported by the findings presented in the article?

Partly

If any results are presented, are all the source data underlying the results available to ensure full reproducibility?

Yes

Are sufficient details provided to allow replication of the method development and its use by others?

Partly

Reviewer Expertise:

Mathematical epidemiology and computational statistics.

I confirm that I have read this submission and believe that I have an appropriate level of expertise to confirm that it is of an acceptable scientific standard, however I have significant reservations, as outlined above.

# Response

**Published:** 2021-01-31

**Authors:** Adeel Razi

**Affiliations:** Monash University, Australia

Dear Dr. Zarebski,

 We would like to thank you for the time and effort you have spent reviewing our manuscript. Below are the replies to the comments. We hope these revisions are what you had in mind.

- *The authors should be commended for the substantial efforts put into making this analysis reproducible with good documentation and by referencing the commit of the JHU repository for the data they used. Although on a fresh installation of Octave version 4.0.0 I was not able to reproduce the computation, even after following the Octave specific instructions regarding the `importdata` function. The data appeared to be read in successfully, but subsequently, an indexing error was hit, and I was not able to debug further. Please note that I have not used Octave/MATLAB for several years so there may have been something obvious I was missing.*

We have tested our code with Octave 5.2 (as mentioned in the paper under software availability) for which it worked however there was a slight mistake in importdata command which is now fixed. OCTAVE 4.0.0 is relatively old hence we cannot guarantee code execution due to backward compatibility issues.

- *The text is very conversational, which is pleasant to read, but which makes it difficult to get a strong grasp on the details. I think it would be much easier to understand the work if the text was heavily edited for brevity.*

We have tried to be slightly more concise in our revisions.

- *The model reduction has left a model in which there is only migration between New York and the other states. I worry that result may be driven entirely by the assumed initial condition in which the epidemic is only seeded in New York (a dubious assumption). I would like to see that the connectivity is robust to changes in the initial condition as I suspect allowing for importation into all the considered states will affect this.*

Initial conditions and other prior assumptions are clearly important aspects. We now try to make this clear by adding the following (just above Equation 1.3):

 “Here we note that the model reduction identified a model where migration was restricted to New York and remaining states. A natural question is whether this reflects the assumption that the epidemic was seeded in New York. This kind of question suggests a new model space in which models that have multiple seeds are compared. More generally, any initial condition or prior assumption of this sort is part of the generative model and can be evaluated using Bayesian model comparison. The application of Bayesian model reduction in this setting would require a full model in which the epidemic was seeded in multiple regions. Reduced models with then comprise seeds in subsets of States. We do not pursue this analysis here but note that this is an interesting question.”

- *I could not see whether it was the weekly number of cases/deaths or the weekly cumulative number of cases/deaths that were being used. The use of cumulative counts is questionable if one is modelling the observations as conditionally independent. Having a more explicit definition of the likelihood function would probably resolve my misunderstanding. For details of the model (before the inclusion of spatial structure) the reader is directed to a previous manuscript which seemed to suggest it was not the cumulative counts used, but then the cumulative counts are frequently referenced in the text as the data that the model is being compared to.*

Models were fit to daily (not cumulative) deaths and positive tests. We have amended the third sentence in the paragraph headed “Dynamic causal modelling” to read *“This furnishes a 4^4 = 256-compartmental model that allows for different combinations of states to generate daily case and death data”.* We have also amended the legend for Figure 3 to “... *explain timeseries (daily death and positive test*) *data …*”. Note that in some sections of the paper we use cumulative deaths as a measure of overall impact and consider its sensitivity to various model parameters. However, the model itself is fit to the daily data.

- *There are comments regarding the limitations of the work scattered throughout the text. I think it would be useful to have a couple of paragraphs towards after the results section spelling these out in one place. In particular, the results "are just model predictions and should not be taken literally. They should be read as the kind of predictions that can be made with suitable modelling."*

We have included a brief paragraph (in the Conclusions section) phrasing some of the key limitations of dynamic causal modelling and variational Bayes:

 “We have noted some of the limitations of dynamic causal modelling—and more generally variational Bayes—above. Two key limitations are (i) the overconfidence problem and (ii) the robustness of the Laplace assumption. The overconfidence problem derives from a failure to explicitly account for conditional dependencies among the factors that constitute the mean field approximation that defines variational procedures. This is a ubiquitous problem in any variational approach that tries to simplify model inversion by breaking the problem into a series of marginal posteriors. Generally, one just acknowledges the overconfidence problem or, as in this application of dynamic causal modelling, inflate the credible intervals such that they encompass the data points. The robustness of the Laplace assumption speaks to the discrepancy between the form of the true and approximate posterior that has two implications. First, one is committed to unimodal Gaussian approximations to the posterior density that might be multimodal. The second implication is that the assumed (quadratic) form for the objective function may be violated, creating local minima. The solution to these problems generally rests on finessing nonlinearities by using different functional forms for the likelihood or priors. For example, using log transforms as in the current application. The very existence of local minima and failures of convergence are often used as a diagnostic to suggest that the generative model (and implicit formal priors) need attention. As illustrated above, different generative models can then be optimized in relation to their free energy.”

- *In my (very limited) experience with variational Bayes methods, I have found the technique to be fiddly. I would like to see more evidence to suggest that the Laplace approximation is reasonable in this setting (i.e., that there is sufficient information to have a posterior that is approximately Gaussian). Ideally, one would re-run some of the inference using something such as MCMC to check that the resulting posterior samples conform to something vaguely Gaussian. Are there some sort of diagnostics that could be applied here?*

We have now added (in the Introduction):

 “Technically, dynamic causal modelling is the application of variational Bayes to state space models of any form; usually, with the objective of inferring the model parameters and structure via Bayesian model inversion and reduction, respectively. Generally, variational Bayes can be an involved process that requires the specification of conjugate priors and variational updates in a model-specific fashion. However, dynamic causal modelling uses a generic scheme called Variational Laplace. Variational Laplace can be used for any generative model because it dispenses with the usual variational updates and uses a gradient descent directly on variational free energy, under the assumption that the posterior is a Gaussian. With suitable transformations of parameters – such as log transforms – this is usually a mild assumption. This is usually licensed by reference to Monte Carlo Markov Chain schemes that relax the Laplace assumption. For state space models of the kind used in this report, the Laplace assumption can be taken as appropriate (Chumbley et al., 2007; Penny et al., 2003; Sengupta et al., 2015)”

**References:**

 Chumbley, J.R., et al., 2007. A Metropolis-Hastings algorithm for dynamic causal models. Neuroimage. 38 **,** 478-87.

 Penny, W., Kiebel, S., Friston, K., 2003. Variational Bayesian inference for fMRI time series. Neuroimage. 19 **,** 727-41.

 Sengupta, B., Friston, K.J., Penny, W.D., 2015. Gradient-free MCMC methods for dynamic causal modelling. Neuroimage. 112 **,** 375-381.

 Sokolov, A.A., et al., 2019. Linking structural and effective brain connectivity: structurally informed Parametric Empirical Bayes (si-PEB). Brain structure & function. 224 **,** 205-217.
